# Hyperglycemia and blood glucose deterioration are risk factors for severe COVID‐19 with diabetes: A two‐center cohort study

**DOI:** 10.1002/jmv.27556

**Published:** 2022-01-08

**Authors:** Fen Xiao, Ying‐Chu Zhou, Mei‐Biao Zhang, Dong Chen, Shao‐Lin Peng, Hao‐Neng Tang, Long Li, Chen‐Yi Tang, Ji‐Yang Liu, Bo Li, Hou‐De Zhou

**Affiliations:** ^1^ National Clinical Research Center for Metabolic Diseases, Hunan Provincial Key Laboratory for Metabolic Bone Diseases, and Department of Metabolism and Endocrinology The Second Xiangya Hospital of Central South University Changsha Hunan China; ^2^ The First Hospital of Changsha, The Hospital of Infectious Diseases of Changsha The Public Health Treatment Center of Changsha Changsha Hunan China; ^3^ The First People's Hospital of Huaihua Huaihua Hunan China

**Keywords:** blood glucose control, COVID‐19, hyperglycemia, risk factors, severe cases

## Abstract

We aimed to assess whether blood glucose control can be used as predictors for the severity of 2019 coronavirus disease (COVID‐19) and to improve the management of diabetic patients with COVID‐19. A two‐center cohort with a total of 241 confirmed cases of COVID‐19 with definite outcomes was studied. After the diagnosis of COVID‐19, the clinical data and laboratory results were collected, the fasting blood glucose levels were followed up at initial, middle stage of admission and discharge, the severity of the COVID‐19 was assessed at any time from admission to discharge. Hyperglycemia patients with COVID‐19 were divided into three groups: good blood glucose control, fair blood glucose control, and blood glucose deterioration. The relationship of blood glucose levels, blood glucose control status, and severe COVID‐19 were analyzed by univariate and multivariable regression analysis. In our cohort, 21.16% were severe cases and 78.84% were nonsevere cases. Admission hyperglycemia (adjusted odds ratio [aOR], 1.938; 95% confidence interval [95% CI], 1.387–2.707), mid‐term hyperglycemia (aOR, 1.758; 95% CI, 1.325–2.332), and blood glucose deterioration (aOR, 22.783; 95% CI, 2.661–195.071) were identified as the risk factors of severe COVID‐19. Receiver operating characteristic (ROC) curve analysis, reaching an area under ROC curve of 0.806, and a sensitivity and specificity of 80.40% and 68.40%, respectively, revealed that hyperglycemia on admission and blood glucose deterioration of diabetic patients are potential predictive factors for severe COVID‐19. Our results indicated that admission hyperglycemia and blood glucose deterioration were positively correlated with the risk factor for severe COVID‐19, and deterioration of blood glucose may be more likely to the occurrence of severe illness in COVID‐19.

## Highlights

1. High fasting blood glucose level, especially the initial level at admission was an important risk factor for severe cases.
2. Deterioration of blood glucose may be more likely to the occurrence of severe 2019 coronavirus disease.

## 1 INTRODUCTION

An unknown cause of pneumonia that has swept the world has been named the 2019 coronavirus disease (COVID‐19) by the World Health Organization.^1^ The virus that caused the outbreak has been identified as the new severe acute respiratory syndrome coronavirus 2 (SARS‐CoV‐2), which is widely distributed in humans and other mammals.^2^, ^3^

With the prevalence of the disease, COVID‐19 patients with diabetes have received a lot of attention. In a nationwide case analysis of 1590 COVID‐19 patients, it was found that a lot of complications, including diabetes, were the most likely potential causes of poor prognosis.^4^ Other studies have also found that chronic diseases such as hypertension and diabetes significantly increase the risk of severe COVID‐19.^5^, ^6^, ^7^ In addition, among 26 death reports caused by COVID‐19, 42.30% of deaths were found to be related to the presence of diabetes.^8^ In contrast, a report of 72 314 COVID‐19 cases showed that compared with subjects without diabetes, the mortality rate of subjects with diabetes increased (7.30% vs. 2.30%).^9^ Moreover, in a recent study of influenza virus infection in mice, Liu et al. found that the specific glucose metabolic pathway is needed to activate interferon regulatory factor‐5 (IRF5)‐induced cytokine production in both cell and mice. This pathway is crucial for an immune response disorder that kills many people with infectious diseases, including those with COVID‐19.^10^ These findings suggest that people with diabetes are more likely to develop serious complications and die of influenza (including COVID‐19) and other infections.

However, the impact of blood glucose, especially uncontrolled blood glucose, on the severity of patients with COVID‐19 is unclear. In the analysis of 11 studies evaluating biochemical abnormalities in patients with COVID‐19, no correlation was found between blood glucose levels and disease severity.^11^ Conversely, there are two studies on the severity and mortality of diabetic patients and found that patients with poorly controlled hyperglycemia (blood glucose > 180 mg/dl) have significantly higher levels of poor prognostic markers than patients with well‐controlled COVID‐19.^12^, ^13^ Besides, in previous SARS and influenza pandemic studies, it was also found that uncontrolled blood glucose was significantly associated with disease severity and mortality in infected patients.^14^, ^15^ For example, the fasting plasma glucose level on admission in patients with influenza A (H1N1) was significantly correlated with the severity of the disease.^16^ Moreover, hyperglycemia was a predictor of the prognosis of various diseases, especially in patients with severe forms of diseases.^17^, ^18^ Recent studies have also shown that avoiding hyperglycemia and controlling the stability of blood glucose can reduce mortality in severe patients.^19^

However, in hospitalized COVID‐19 patients, the control of blood glucose stability is easily overlooked because the vast majority of hospitalized COVID‐19 patients require fluid infusion, and improper infusion of fluids containing glucose can cause blood glucose instability, which is not yet clear as to whether it is related to the severity of COVID‐19. Therefore, the purpose of this study was to find out whether the severity in patients with COVID‐19 has any correlation to the changes of blood glucose levels, to improve the management of diabetic patients with COVID‐19.

## 2 MATERIALS AND METHODS

### 2.1 Study design and participants

The Ethics Committee (LYF2020115) of the National Clinical Research Center for Metabolic Diseases of the Second Xiangya Hospital approved the study. All patients enrolled in this two‐center cohort study were diagnosed with the 2019 novel coronavirus infection with pneumonia (NCIP) according to the guideline of the National Health Commission of China,^20^ and the diagnoses were confirmed by a panel of experts. Patients with NCIP who were admitted continuously from January 1 to February 19, 2020, were included in the study after obtaining their consent. After the diagnosis of COVID‐19, the fasting blood glucose (FBG) levels were followed up on the 1st day after admission (initial blood glucose), the average blood glucose at the 8th and 9th day after admission (mid‐term blood glucose) and at discharge, and the clinical data and laboratory results were also collected, the patients were followed from admission to discharge for the FBG levels and severity of the COVID‐19. We collected a total of 279 confirmed cases of COVID‐19, included 241 COVID‐19 patients who agreed to the FBG measurements, and excluded 38 patients who did not agree with blood glucose measurement.

### 2.2 Definitions

According to the guideline of the National Health Commission of China,^20^ a confirmed case was defined as a suspected case whose laboratory COVID‐19 respiratory tract specimen was positive by a real‐time reverse transcription‐polymerase chain reaction test, the severe disease was defined as a confirmed case with one of the following symptoms: (1) shortness of breath, respiratory rate ≥30 times/min; (2) at rest oxygen saturation, ≤93%; and (3) arterial partial oxygen pressure (PaO_2_)/oxygen absorption concentration (FiO2), ≤300 mmHg (1 mmHg = 0.133 kPa). Diabetes mellitus and other comorbidities were confirmed by reviewing patients' medical records.

According to the diagnostic criteria of metabolic syndrome, FBG higher than 6.1 mmol/L was considered hyperglycemia,^21^ and the glycemic recommendations for nonpregnant adults with diabetes from American Diabetes Association (4.0–7.2 mmol/L),^22^ the admission hyperglycemia cohort was divided into three groups: good blood glucose control group (defined as mid‐term FBG lower than 6.1 mmol/L), fair blood glucose control group (defined as the changes of mid‐term blood glucose levels related to initial blood glucose levels <1.1 mmol/L), and blood glucose deterioration (defined as the changes of mid‐term blood glucose levels related to initial blood glucose levels ≥1.1 mmol/L).

### 2.3 Data collection

The research teams of the First Hospital of Changsha and the First Hospital of Huaihua extracted and analyzed the data from the patients' electronic medical records. Data on patients' epidemiological, demographic, and clinical characteristics, laboratory parameters, and outcomes were measured using standardized instruments. The collected data included: age, gender, height, weight, exposure history, history of diabetes, and potential complications (e.g., hypertension, cardiovascular disease, cerebrovascular disease, chronic respiratory disease, chronic kidney disease, chronic liver disease, and malignant tumor); symptoms on admission to the hospital (e.g., fever, cough, shortness of breath, and myalgia); laboratory parameters on admission (e.g., blood glucose, creatine kinase [CK], erythrocyte sedimentation rate [ESR], lactate dehydrogenase [LDH], and total lymphocyte count); and medical treatment (e.g., antiviral therapy, glucocorticoid therapy, mechanical ventilation, and insulin hypoglycemia). All data were reviewed by a team of experienced doctors.

### 2.4 Statistical analysis

Categorical variables (gender, exposure history, signs, and symptoms at admission, comorbidities) were calculated as frequencies and percentages (with the available data) and continuous variables (age, height, weight, BMI, courses, laboratory parameters) were presented as mean or median with the standard deviation or interquartile range (IQR). We used univariate and multivariate logistic regression to explore the risk factors associated with severe COVID‐19 and reported the odds ratio (OR) and 95% confidence interval (95% CI). Multiple independent variables were adjusted in the multivariate logistic regression model. For the logistic regression model where the independent variables are ordered multi‐classification variables, we treat the ordered multi‐classification variables as dumb variables, and each level is compared with the first level. In all the multiple regression models established by adjusting some variables, the test efficiency of the model is more than 80%. The receiver operating characteristic (ROC) curve was used for diagnosis analysis. SPSS (version 25.0) was used for the statistical analyses. A two‐sided *p* < 0.05 was considered statistically significant.

## 3 RESULTS

### 3.1 Baseline and clinical characteristics of the patients with COVID‐19

Among the study's 241 patients, 21.16% were severe cases and 78.84% were nonsevere cases (Table 1). Their median age was 45.0 years (IQR, 34.0–58.0), 51.04% were female, and 48.96% were male. With respect to their exposure history, 43.99% had been to Wuhan, 47.30% had close contact with confirmed cases from Wuhan, and 8.71% had no contact history. The median length of hospital stay was 18.15 days, and the onset days before admission was 5.00 days. The most common COVID‐19 symptoms were fever, cough, fatigue, and shortness of breath. The most observed comorbidities were hypertension, diabetes, fatty liver, chronic liver disease, cardiovascular disease. The least common comorbidities were cancer, chronic kidney disease, digestive tract disease, emphysematous bullae, infections, and nervous system disease (Table S1).

**Table 1:** Baseline, demographic, and clinical characteristics of patients infected with COVID‐19

Characteristic	All patients (*N* = 241）	*p*	*p*‐value adjusted for age, gender, and BMI	
			OR (95% CI)	*p*
Age (years)	45.0 (34.0–58.0)	<0.001	‐	‐
<45	119 (49.38%)			
≥45	122 (50.62%)			
Gender		0.340	‐	‐
Male	118 (48.96%)			
Female	123 (51.04%)			
BMI	23.20 (21.10–25.60)	0.017	‐	‐
Exposure history				
Contact with confirmed case from Wuhan	114 (47.30%）	0.026	2.452 (1.191–5.050)	0.015
Course				
Onset days before on admission	5.00 (3.00–8.00）	0.235	1.027 (0.972–1.085)	0.341
Hospital stay	18.15 (8.94）	0.002	1.048 (1.011–1.086)	0.011
Severe case	51 (21.16%)	‐	‐	‐
Signs and symptoms at admission				
Fever	160 (66.39%)	0.001	6.072 (2.302–16.020)	<0.001
Cough	138 (57.26%)	0.015	2.680 (1.257–5.714)	0.011
Shortness of breath	30 (12.45%)	<0.001	4.738 (2.007–11.186)	<0.001
Myalgia	23 (9.54%)	0.099	2.192 (0.797–6.024)	0.128
Fatigue	80 (33.20%)	<0.001	3.142 (1.578–6.256)	0.001
Comorbidities				
Fatty liver	8 (3.32%)	0.058	3.833 (0.820–17.918)	0.088
Diabetes mellitus	18 (7.47%）	0.477	0.486 (0.143–1.647)	0.246
Hypertension	31 (12.86%)	<0.001	3.371 (1.393–8.158)	0.007
Cardiovascular disease	7 (2.90%)	0.033	2.310 (0.459–11.639)	0.310
Emphysematous bullae	2 (0.83%)	0.999	‐	‐
Cerebral infarction	6 (2.49%)	0.006	12.953 (2.641–63.534)	0.002
Digestive tract disease	1 (0.41%)	1.000	‐	‐
Chronic kidney disease	1 (0.41%)	0.787	1.557 (0.269–9.006)	0.621
Chronic respiratory diseases	3 (1.24%)	0.098	4.605 (0.388–54.666)	0.226
Laboratory parameter				
d‐dimer (µg/ml; normal range 0–1)	0.43 (0.17–0.68)	0.027	1.188 (0.963–1.467)	0.108
Increased	17 (7.05%)			
Lactate dehydrogenase (U/L; normal range 135–225)	179.30 (143.20–196.10)	<0.001	1.015 (1.008–1.021)	<0.001
Increased	41 (17.01%)			
Decreased	48 (19.90%)			
Creatine kinase (U/L; normal range 10–190)	83.40 (53.40–103.00)	0.012	1.004 (1.001–1.007)	0.021
Increased	15 (6.22%)			
Decreased	57 (23.65%)			
Albumin (g/L; normal range 35–55)	37.93 (35.61–40.21)	<0.001	0.815 (0.734–0.904)	<0.001
Decreased	44 (18.25%)			
Aspartate aminotransferase (U/L; normal range 0–37)	26.28 (20.29–28.50)	0.001	1.034 (1.003–1.066)	0.032
Increased	30 (12.45%)			
Alanine aminotransferase (U/L; normal range 0–42)	22.01 (15.57–26.38)	0.097	1.02 (0.99–1.05)	0.150
Increased	16(6.64%)			
Erythrocyte sedimentation rate (mm/h; normal range 0.0–15.0)	21.23 (7.40–25.20)	<0.001	1.045 (1.026–1.064)	<0.001
Increased	146 (60.58%)			
C‐reactive protein (mg/L; normal range 0.0–8.0)	39.65 (28.00–39.65)	0.005	1.010 (0.995–1.026–1.03)	0.199
Increased	230 (95.44%)			
Lymphocytes (×10^9^/L; normal range 0.8–4.0)	1.26 (0.91–1.49)	<0.001	0.883 (0.838–0.931)	<0.001
Increased	2 (0.83%)			
Decreased	44 (18.26%)			
Lymph% (normal range 20–40）	27.78 (22.00–31.40)	<0.001	0.104 (0.038–0.286)	<0.001
Increased	24 (9.96%)			
Decreased	48 (19.92%)			
White blood cell count (×10^9^/L; normal range 4–10)	4.77 (3.59–5.48)	0.242	0.867 (0.694–1.084)	0.211
Increased	3 (1.24%)			
Decreased	78 (32.37%)			

*Note*: Data are expressed as median (interquartile range), standard deviation, *n* (%), or *n*/*N* (%), where *N* is the total number of cases. The model was adjusted by gender, age, and BMI. A *p* < 0.05 was considered statistically significant.

Abbreviations: BMI, body mass index; CI, confidence interval; COVID‐19, 2019 coronavirus disease; OR, odds ratio.

### 3.2 Predictors of severity among COVID‐19 patients

#### 3.2.1 Risk factors for severe COVID‐19

The regression analysis of risk factors severe COVID‐19 is presented in Table 1. After adjusting for gender, age, and BMI, patients with hypertension (OR, 3.371; 95% CI, 1.393–8.158), fever (OR, 6.072; 95% CI, 2.302–16.020), cough (OR, 2.680; 95% CI, 1.257–5.714), shortness of breath (OR, 4.738; 95% CI, 2.007–11.186), and fatigue (OR, 3.142; 95% CI, 1.578–6.256) were found to be more prone to severe symptoms than patients without these risk factors. However, there was no significant difference between the presence or absence of diabetes and severe COVID‐19. The initial (OR, 1.627; 95% CI, 1.342–1.973) and mid‐term (OR, 1.599; 95% CI, 1.349–1.895) blood glucose levels were related to markers of infection, such as C‐reactive protein (CRP) (OR, 1.010; 95% CI, 0.995–1.026) and the ESR (OR, 1.045; 95% CI, 1.026–1.064). Serum aspartate aminotransferase (AST) (OR, 1.034; 95% CI, 1.003–1.066), albumin (OR, 0.815; 95% CI, 0.734–0.904), LDH (OR, 1.015; 95% CI, 1.008–1.021), and blood lymphocyte count (OR, 0.883; 95% CI, 0.838–0.931) were associated with severe cases. The results of the unadjusted analyses are also presented (Table S1), which is consistent with the above‐mentioned results with adjusted analyses.

#### 3.2.2 Regression analysis of blood glucose levels and severe COVID‐19

The results of the univariate and multivariable analysis of blood glucose levels and severe COVID‐19 are summarized in Table 2. In this cohort (*n* = 241), the initial (aOR, 1.938; 95% CI, 1.387–2.707) and mid‐term blood glucose level (aOR, 1.758; 95% CI, 1.325–2.332) were identified as the risk factors of severe cases. The blood glucose level at discharge was consistent with the results of the regression analysis and had no effect on the occurrence of severe cases. After various adjustments (Model Ⅲ and Ⅳ) were made (Table S2), the results were almost the same as when they were unadjusted. More importantly, in those cases whose FBG > 6.1 mmol/L, the initial (*n* = 87) and mid‐term (*n* = 107) hyperglycemia had significant associations with the severity of COVID‐19 (Table S3).

**Table 2:** Univariate and multivariate regression analysis of the relationship between three fasting blood glucose levels and severe COVID‐19

Variable (mmol/L)	All patients (*N* = 241）	Univariate analysis			Multivariable analysis		
		OR	95% CI	*p*	OR	95% CI	*p*
Initial blood glucose	5.50 (4.81–6.91)	1.693	1.415–2.026	<0.001	1.938	1.387–2.707	<0.001
Mid‐term blood glucose	5.79 (4.89–7.58)	1.695	1.441–1.994	<0.001	1.758	1.325–2.332	<0.001
Blood glucose at discharge	5.24 (4.73–6.52)	1.231	1.057–1.434	0.001	1.160	0.864–1.557	0.323

*Note*: Multivariable analysis model was adjusted for gender, age, BMI, diabetes, hypertension, fatty liver, cardiovascular disease, chronic respiratory disease, emphysematous bullae, cerebral infarction, digestive tract disease, nervous system disease, endocrine disease, chronic liver disease, chronic kidney disease, cancer, infection, fever, cough, poor appetite, shortness of breath, myalgia, headache, dizziness, diarrhea, fatigue, nausea/vomiting, pharyngalgia, runny nose, lymphocyte percentage (%), lymphocyte count (×10^9^/L), C‐reactive protein (mg/L), erythrocyte sedimentation rate (mm/h), aspartate aminotransferase (U/L), albumin (g/L), creatine kinase (U/L), lactate dehydrogenase (U/L), and d‐dimer (μg/L).

Abbreviations: BMI, body mass index; CI, confidence interval; COVID‐19, 2019 coronavirus disease; OR, odds ratio.

#### 3.2.3 The value of blood glucose ROC curve to distinguish severe and nonsevere case

**Figure 1:**
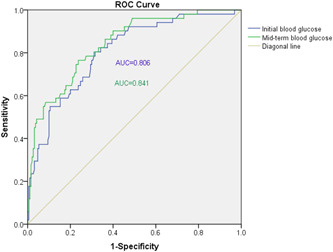
Receiver operating characteristic (ROC) curve for evaluating blood glucose levels as predictors on distinguishing severe and nonsevere cases. The area under ROC curve (AUC) was 0.806 and 0.841, (95% confidence interval [95% CI] = 0.739–0.872, *p* < 0.001) and (95% CI = 0.782–0.901, *p* < 0.001), with a sensitivity of 80.40%, 76.50% and a specificity of 68.40%, 76.30%, respectively

ROC curve analysis was performed to evaluate the specificity and sensitivity of Initial blood glucose and mid‐term blood glucose levels on distinguishing severe and nonsevere cases. As shown in Figure 1, for the Initial blood glucose, the optimal cut‐off point was 5.83, providing the specificity and sensitivity of 68.40% and 80.40%, respectively. For the mid‐term blood glucose, the optimal cut‐off point was 6.89, providing the specificity and sensitivity of 76.30% and 76.50%, respectively. Besides, the former's area under ROC curve (AUC) was 0.806 and the latter was 0.841, indicating hyperglycemia was likely to be a strong risk factor for predicting severe cases.

#### 3.2.4 The relationship between blood glucose deterioration and severe COVID‐19

In COVID‐19 patients with hyperglycemia, multivariate regression analysis (Table 3) showed that patients with the blood glucose deterioration (the increase of mid‐term blood glucose levels ≥1.1 mmol/L) are more likely to be severe COVID‐19 than patients with good blood glucose control (aOR, 22.783; 95% CI, 1.661–195.071) after being adjusted for gender, age, BMI, hypertension, diabetes, fever, cough, lymphocyte count, CRP, ESR, fatty liver, LDH, and d‐dimer, even if unadjusted or partially adjusted, patients with the blood glucose deterioration are also more severe cases. In the fair blood glucose control group (Table 3), whose increase of mid‐term blood glucose levels lower than 1.1 mmol/L, there is no significant correlation between this lightly blood glucose change and the occurrence of severe COVID‐19, although the result shows a tendency of correlation (*p* = 0.058).

**Table 3:** Multivariate regression analysis of the relationship between different blood glucose control and severe COVID‐19 with hyperglycemia at admission (*n* = 87)

Models	Groups	*p*	OR (95% CI)
Model I	Good blood glucose control	0.003	
	Fair blood glucose control	0.041	4.141 (1.060–16.174)
	Blood glucose deterioration	0.001	13.571 (2.991–61.586)
Model II	Good blood glucose control	0.006	
	Fair blood glucose control	0.058	3.783 (0.954–14.992)
	Blood glucose deterioration	0.002	11.755 (2.527–54.690)
Model III	Good blood glucose control	0.011	
	Fair blood glucose control	0.174	3.965 (0.544–28.897)
	Blood glucose deterioration	0.005	20.386 (2.528–164.417)
Model IV	Good blood glucose control	0.010	
	Fair blood glucose control	0.205	3.715 (0.489–28.225)
	Blood glucose deterioration	0.004	22.783 (2.661–195.071)

*Note*: A total of 87 people with initial blood glucose hyperglycemia were included in this model. Model I is the unadjusted model. Model II was adjusted for gender, age, BMI. Model III was adjusted for Model II and hypertension, diabetes, fever, cough, lymphocyte count (×10^9^/L), C‐reactive protein (mg/L), erythrocyte sedimentation rate (mm/h). Model IV was adjusted for Model III and fatty liver, Lactate dehydrogenase (U/L), d‐dimer (µg/ml).

Abbreviations: BMI, body mass index; CI, confidence interval; COVID‐19, 2019 coronavirus disease; OR, odds ratio.

## 4 DISCUSSION

The blood glucose management of COVID‐19 patients with diabetes is very important but it is not easy to manage well, as most of the hospitalized patients received intravenous drip to replenish energy which leads to the blood glucose fluctuations, and it is not clear whether the blood glucose fluctuations affect the severity of the COVID‐19 patients. Our study found that high FBG level, especially the initial level at admission and blood glucose deterioration were likely to be an important risk factor for severe cases using various logistic regression models and stratification and interaction testing (Tables 2 and 3). Our findings were also consistent with current basic studies, such as the finding that the functional receptor (angiotensin‐converting enzyme 2, ACE2) of SARS‐CoV‐2 is expressed in islet cells, SARS‐CoV‐2 can use ACE2 effectively to destroy islet cells, resulting in an islet‐function damage, hyperglycemia or deterioration of blood glucose in patients with diabetes.^23^, ^24^ In view of the peculiarity and complexity of COVID‐19, some potential comorbidities might have significant effects on the prognosis of patients with COVID‐19, while the proportion of patients with diabetes mellitus in severe cases and deaths from COVID‐19 was high, making it more difficult to treat them. Therefore, the control of blood glucose in patients with hyperglycemia or diabetes is particularly important in the comprehensive treatment of COVID‐19. Not surprisingly, the blood glucose level at discharge, controlled blood glucose group, and poor blood glucose group in the occurrence of severe disease had no effect on severe cases, most likely because the level had been controlled after treatment in the hospital.

We also found that the initial and mid‐term blood glucose levels were related to markers of infection, such as CRP and the ESR (Table 1), which indicates that hyperglycemia might be associated with the release of more inflammatory cytokines. As we know, the release of a large number of inflammatory cytokines can lead to inflammatory cytokine storm,^25^ which directly leads to the occurrence of severe COVID‐19.^26^ Therefore, for COVID‐19 patients with diabetes mellitus, it is very important to maintain blood glucose control within a stable and normal range.

However, it has been inconclusive whether the proportion of COVID‐19 patients with diabetes is higher than that of the general population. In a previous research of 99 patients by Chen et al.^27^ and another of 41 patients by Huang et al.,^28^ the prevalence of diabetes was higher than that of the general population age 40–59 years (11.5%). Furthermore, Wang et al.^29^ and Chen et al.^27^ reported the prevalence of diabetes in 138 and 99 COVID‐19 patients, respectively, was close to the prevalence in the general population between 40 and 59 years old. However, the prevalence of diabetes in our sample with COVID‐19 was only 7.47%, which was far lower than the prevalence in the Wuhan and general population in the early stage of the epidemic.^30^ This result also appears in data analysis of 1099 cases from a large sample of the whole country.^31^ The reason might be most of the severe patients are middle‐aged and elderly people, especially those with diabetes, they had less opportunities to travel to Wuhan or have more prevention and isolation, but whether this is related to the effective isolation measures taken in China during the outbreak still need a validation. Confusingly, unlike previous studies, diabetes was not found to be a risk factor for severe COVID‐19 in our logistic regression analysis. A possible reason is that the number of diabetic patients with COVID‐19 was small, and there may have regional differences in the occurrence of diabetes.^24^ If possible, larger sample sizes will be needed to confirm this.

Based on our descriptive statistics, in all the COVID‐19 patients in Changsha, 43.99% had been to Wuhan and 47.30% had close contact with the confirmed cases coming from Wuhan, only 8.71% had no contact history. These data are extremely important to assess the effectiveness of epidemic prevention measures, as Changsha is adjacent to Wuhan, which is only an hour and a half away by high‐speed rail. There are a population of more than 13 million people and a large population mobility in Changsha and Huaihua city, but only 279 people have been diagnosed with COVID‐19. Of the 241 people included in this study, 106 were directly from Wuhan, and they may have infected 114 people in the absence of adequate public awareness. With the improvement of understanding of COVID‐19 and disease control measures (e.g., wearing a mask, home quarantine), the people with the subsequent infection were only 21, epidemic control of COVID‐19 has been achieved, which illustrate our prevention and control measures are very effective. This has important reference and guiding significance for the areas where the epidemic is still widespread. Besides, 21.16% of patients in the study had severe COVID‐19, which was consistent with previous research.^31^, ^32^ Fever, cough, fatigue, and shortness of breath were the most common symptoms on admission to the hospital, which was consistent with the findings of several early studies on the clinical characteristics of the 2019 NCIP in China.^29^, ^33^ In our study, the most common laboratory abnormalities observed in severe COVID‐19 were blood glucose, CK, AST, ESR, LDH, and total lymphocyte count. These abnormalities suggest that COVID‐19 may be related to cellular immunodeficiency, myocardial damage, and liver damage, thus, reflecting the functions of different organs and systems, which were similar to those previously observed in patients infected with Middle East respiratory syndrome coronavirus (MERS‐CoV) and SARS‐CoV.^5^, ^34^

A limitation of this study is that there were fewer patients with diabetes, cardiovascular disease, chronic respiratory disease, and other comorbidities, which might have biased the results of the analysis on the impact of comorbidities, such as diabetes, on the severity of COVID‐19 in critical patients. Second, more detailed patient information, particularly regarding clinical outcomes, was unavailable at the time of analysis. However, the data in this study permit an early assessment of the relationship of blood glucose levels, blood glucose control status, and severe COVID‐19.

In conclusion, our results indicated that admission hyperglycemia was positively correlated with the risk factor for severe COVID‐19, and blood glucose deterioration may be more likely to the occurrence of severe illness in COVID‐19. Therefore, for COVID‐19 patients with diabetes mellitus, it is very important to maintain blood glucose control within a stable and normal range.

## CONFLICT OF INTERESTS

The authors declare that there are no conflict of interests.

## ETHICS STATEMENT

The studies involving human participants were reviewed and approved by the Ethics Committee of the Second Xiangya Hospital, Central South University.

## AUTHOR CONTRIBUTIONS

Hou‐De Zhou signed the study and reviewed/edited the manuscript. Fen Xiao wrote the manuscript and performed the data analysis. Ying‐Chu Zhou performed the follow‐up and the data analysis. Bo Li, Mei‐Biao Zhang, and Shao‐Lin Peng provided data. Dong Chen and Hao‐Neng Tang contributed to statistical analysis. Long Li, Chen‐Yi Tang, and Ji‐Yang Liu checked the data.

## Supporting information

Supporting information.

## Data Availability

Please contact the corresponding author for data requests.
