# Supplementary material for: Hyperglycemia and blood glucose deterioration are risk factors for severe COVID‐19 with diabetes: A two‐center cohort study
Source: J Med Virol. 2022 Jan 8;94(5):1967–75. doi: 10.1002/jmv.27556 (PMC9015512; doi:10.1002/jmv.27556)
Supplement: Supplementary file 1 — Supporting information. [file JMV-94-1967-s001.pdf]

Supplementary Materials:

Supplementary Table S1: Analysis of the risk factors for severe SARS-CoV-2 infection.

Variable	OR (95% CI)	*p*
Age (years)	1.056(1.033-1.080)	<0.001
Gender	1.353(0.727-2.516)	0.340
BMI	1.117(1.020-1.223)	0.017
Exposure history		
Contact with a confirmed case	0.479(0.251-0.916)	0.026
Unclear history	2.000 (0.762-5.253)	0.159
Course (days)		
Onset of illness to hospitalization	1.031(0.980-1.084)	0.235
Length of hospital stay	1.055(1.020-1.092)	0.002
Comorbidity		
Hypertension	6.286(2.833-13.947)	<0.001
Diabetes mellitus	1.480(0.502-4.363)	0.477
Fatty liver	3.957(0.954-16.411)	0.058
Cardiovascular disease	5.305(1.148-24.517)	0.033
Cerebral infarction	20.543(3.343-180.120)	0.006
Other endocrine diseases	3.837(0.527-27.928)	0.184
Chronic liver disease	1.252(0.245-6.395)	0.787
Chronic respiratory disease	7.714(0.685-86.835)	0.098
Signs and symptoms at admission		
Fever	4.891(1.988-12.032)	0.001
Cough	2.329(1.182-4.587)	0.015
Shortness of breath	6.808(3.028-15.304)	<0.001
Myalgia	2.171(0.864-5.450)	0.099
Headache	2.170(0.762-6.182)	0.147
Dizziness	1.218(0.255-5.823)	0.805
Diarrhea	1.471(0.412-5.259)	0.552
Fatigue	3.231(1.708-6.109)	<0.001
Nausea vomiting	1.913(0.230-15.910)	0.549
Pharyngalgia	3.542(0.809-15.518)	0.093
Runny nose	1.247(0.127-12.244)	0.850
Laboratory parameter		
Initial blood glucose	1.693(1.415-2.026)	<0.001
Mid-term blood glucose	1.695(1.441-1.994)	<0.001

Blood glucose at discharge	1.231(1.057-1.434)	0.008
White blood cell count (×10^9/L)	0.889(0.730-1.083)	0.242
Hemoglobin (g/L)	0.997(0.977-1.017)	0.750
Platelet count (×10^9/L)	0.997(0.993-1.002)	0.293
Lymphocyte percentage (%)	0.081(0.031-0.211)	<0.001
Lymphocyte count (×10^9/L)	0.867(0.826-0.911)	<0.001
C-reactive protein (mg/L)	1.020(1.006-1.034)	0.005
Erythrocyte sedimentation rate (mm/h)	1.055(1.037-1.074)	<0.001
Alanine aminotransferase (U/L)	1.020(0.995-1.045)	0.097
Aspartate aminotransferase (U/L)	1.047(1.019-1.076)	0.001
Total bilirubin (μmol/L)	1.023(0.991-1.056)	0.169
Albumin (g/L)	0.782(0.712-0.859)	<0.001
Serum creatinine (μmol/L)	1.002(0.986-1.018)	0.831
Blood urea nitrogen (mmol/L)	1.133(0.967-1.326)	0.122
Creatine kinase (U/L)	1.004(1.001-1.007)	0.012
Creatine kinase–MB (U/L)	1.002(0.983-1.021)	0.849
Lactate dehydrogenase (U/L)	1.017(1.011-1.023)	<0.001
Triglyceride (mmol/L)	0.913(0.609-1.368)	0.658
Total cholesterol (mmol/L)	0.814(0.516-1.284)	0.367
D-dimer (μg/L)	1.260(1.027-1.547)	0.027

OR, odds ratio; CI, confidence interval. A *p*-value < 0.05 was considered statistically significant. The model was unadjusted.

Supplementary Table S2: Multivariate regression analysis of the relationship between three times blood glucose level and severe SARS-CoV-2 infection.

	Exposure	OR (95%CI)	*p*
**Model I**	Initial blood glucose	1.693 (1.415-2.026)	<0.001
	Mid-term blood glucose	1.695 (1.441-1.994)	<0.001
	Blood glucose at discharge	1.231 (1.057-1.434)	0.008
**Model II**	Initial blood glucose	1.627(1.342-1.973)	<0.001
	Mid-term blood glucose	1.599(1.349-1.895)	<0.001
	Blood glucose at discharge	1.097(0.928-1.297)	0.278
**Model III**	Initial blood glucose	1.724 (1.316-2.259)	<0.001
	Mid-term blood glucose	1.620 (1.260-2.083)	<0.001
	Blood glucose at discharge	1.093 (0.837-1.428)	0.512
**Model IV**	Initial blood glucose	2.274 (1.481-3.490)	<0.001
	Mid-term blood glucose	1.926 (1.399-2.650)	<0.001
	Blood glucose at discharge	1.191 (0.831-1.709)	0.341

A total of 241 people were included in the models. OR, odds ratio; CI, confidence interval. Model I is the unadjusted model. Model II was adjusted for gender, age, BMI. Model III was adjusted for Model II and diabetes, hypertension, fatty liver, and cardiovascular disease, cerebral infarction, fever, cough, shortness of breath, fatigue, lymphocyte count (×109/L), C-reactive protein (mg/L), erythrocyte sedimentation rate (mm/h), aspartate aminotransferase (U/L), albumin (g/L), lactate dehydrogenase (U/L), and D-dimer (μg/L). Model IV was adjusted for Model III and contact with confirmed case, emphysematous bullae, digestive tract disease, nervous system disease, endocrine disease, chronic respiratory disease, chronic liver disease, chronic kidney disease, cancer, infection, poor appetite, myalgia, headache, dizziness, diarrhea, nausea/vomiting, pharyngalgia, runny nose, creatine kinase (U/L), lymphocyte percentage (%).

Supplementary Table S3: Multivariate regression analysis of the relationship between hyperglycemia on admission and severe SARS-CoV-2 infection.

	**Exposure**	**OR (95% CI)**	***p***
**Model I**	Initial blood glucose hyperglycemia	5.805 (2.965-11.367)	<0.001
	Mid-term blood glucose hyperglycemia	4.519 (2.181-9.361)	<0.001
**Model II**	Initial blood glucose hyperglycemia	5.105 (1.733-15.036)	0.003
	Mid-term blood glucose hyperglycemia	4.124 (1.277-13.322)	0.018
**Model III**	Initial blood glucose hyperglycemia	4.519 (2.181-9.361)	<0.001
	Mid-term blood glucose hyperglycemia	5.981(2.647-13.512)	<0.001
**Model IV**	Initial blood glucose hyperglycemia	13.124(2.996-57.489)	0.001
	Mid-term blood glucose hyperglycemia	4.574(1.267-16.515)	0.020

A total of 87 people with initial blood glucose hyperglycemia and 107 people with mid-term blood glucose hyperglycemia was included in these models. OR, odds ratio; CI, confidence interval. Model I is the unadjusted model. Model II was adjusted for gender, age, and BMI. Model III was adjusted for Model II and diabetes, hypertension, fatty liver, cardiovascular disease, cerebral infarction, fever, cough, shortness of breath, fatigue, lymphocyte count (× 10^9/L), C-reactive protein (mg/L), erythrocyte sedimentation rate (mm/h), aspartate aminotransferase (U/L), albumin (g/L), lactate dehydrogenase (U/L), and D-dimer (μg/L). Model IV was adjusted for Model III and contact with confirmed case, cerebral infarction, endocrine disease, chronic liver disease, poor appetite, myalgia, headache, dizziness, diarrhea, nausea/vomiting, pharyngalgia, runny nose, creatine kinase (U/L).
